# Association between diabetes mellitus and risk of Alzheimer’s disease: a meta-analysis and systematic review

**DOI:** 10.3389/fendo.2026.1736410

**Published:** 2026-03-03

**Authors:** Ju Wu, Jin Li, Xiaolan Qin, Weiping Chen

**Affiliations:** 1 Chonggang General Hospital, Chongqing, China; 2 Department of Oncology, Army Medical Center of PLA, Chongqing, China; 3 Health Care Department, Yangjiaping Branch of Jiulongpo District People’s Hospital, Chongqing, China

**Keywords:** Alzheimer’s disease, dementia, diabetes mellitus, meta-analysis, systematic review

## Abstract

**Background:**

Diabetes mellitus (DM) is hypothesized to increase the risk of Alzheimer’s disease (AD). However, existing studies have yielded conflicting results, with some demonstrating a significant association between DM and AD risk while others have not. Therefore, this meta-analysis aimed to systematically evaluate the association between DM and AD risk.

**Methods:**

Comprehensive searches were conducted in PubMed, Web of Science, and Embase databases to identify cohort or case–control studies investigating the association between DM and AD risk. All eligible studies published before October 2025 were included. The Newcastle–Ottawa Scale (NOS) was used to assess the risk of bias. Hazard ratio (HR) and 95% confidence interval (CI) were pooled as the effect size for meta-analysis. Heterogeneity among studies was evaluated using Cochran’s *Q* test and *I*^2^ statistics. Statistical analyses were performed with RevMan 5.3 software.

**Results:**

A total of 11 studies involving 3,393,545 participants were included. A meta-analysis revealed that DM was significantly associated with an increased risk of AD (HR = 1.36, 95% CI (1.19, 1.55), *P* < 0.00001). A subgroup analysis showed that DM increased the risk of AD regardless of sample size (<100,000: HR = 1.33, 95% CI (1.11–1.59), *P* = 0.002; >100,000: HR = 1.39, 95% CI (1.13–1.71), *P* = 0.002). The consistency in *P*-values may be coincidental, and the results should be interpreted in conjunction with the high heterogeneity across studies. This association was consistent in both Asian (HR = 1.45, 95% CI: 1.20–1.76, *P* < 0.00001) and non-Asian populations (HR = 1.29, 95% CI: 1.13–1.48, *P* < 0.00001). After adjusting for APOE ϵ4 mutations, there was no statistically significant difference in the risk association between DM and AD (HR = 1.07, 95% CI (0.97–1.19), *P* = 0.177), whereas without adjustment for APOE ϵ4 mutation, DM was associated with an increased AD risk (HR = 1.42, 95% CI (1.23–1.64), *P* < 0.00001).

**Conclusion:**

This meta-analysis provides compelling evidence that DM is an independent risk factor for AD, offering important implications for clinical practice and future research. However, due to the methodological limitations of this study, the results should be interpreted with caution. Large-scale, high-quality prospective cohort studies are needed to fully investigate the relationship between DM and AD risk.

**Systematic review registration:**

https://www.crd.york.ac.uk/prospero/, identifier CRD420251159844.

## Introduction

Diabetes mellitus (DM) is a chronic metabolic disorder characterized by hyperglycemia, primarily classified into type 1 diabetes mellitus (T1DM) and type 2 diabetes mellitus (T2DM) based on etiological differences (1, 2). T1DM results from the autoimmune destruction of pancreatic islet cells, leading to absolute insulin deficiency (3). In contrast, T2DM is caused by relative insulin deficiency and/or insulin resistance in target tissues, resulting in persistent hyperglycemia and subsequent multi-organ damage (4, 5). T2DM accounts for 90%–95% of DM patients, with its prevalence increasing annually. Complications such as diabetic nephropathy and diabetic foot severely impact human health (6).

Dementia is a neurodegenerative disease that progressively damages nerve cells and impairs brain function, leading to deterioration of cognitive abilities (7, 8). As one of the most pressing global public health challenges, dementia imposes a substantial economic burden worldwide (9). Alzheimer’s disease (AD) is the most common form of dementia, accounting for approximately 60%–70% of cases (10). Pathologically, AD is characterized by extracellular amyloid plaques composed of amyloid-beta (Aβ) peptide and intracellular neurofibrillary tangles (NFTs) formed by hyperphosphorylated tau protein (11). Currently, approximately 55 million people worldwide are living with AD, with the number doubling every 5 years (12, 13). It is estimated that by 2050, the global prevalence of AD will reach 152 million, equivalent to one new case every 3 s (12). In the United States, approximately 5.3 million individuals have AD, of whom 5.1 million are 65 years or older (14). In 2019, the number of AD and other dementia cases in China was 13,143,950, accounting for 25.5% of the global total (15), and the number of deaths attributed to these conditions was 320,715, representing 19.8% of global deaths (15). Cognitive impairment in patients with AD is the primary driver of loss of self-care ability, which imposes a substantial socioeconomic burden on societies worldwide. The global socioeconomic cost of dementia was estimated at $957.56 billion in 2015, and projections indicate that this figure will rise to $2.54 trillion by 2030 and further to $9.12 trillion by 2050 (16).

Approximately one-third of AD cases are attributed to modifiable and treatable risk factors, highlighting the importance of early prevention (17). Among these factors, DM has emerged as a key candidate risk factor for AD. Accumulating evidence suggests a complex bidirectional association between DM and AD: DM may induce AD, while pathological products of AD may promote DM progression (18, 19). Both diseases share common pathogenic mechanisms, including insulin resistance, inflammation, and mitochondrial dysfunction. Additionally, DM-related metabolic disturbances may contribute to amyloid formation in AD (19–22).

This bidirectional association, however, remains controversial: some studies have failed to detect a significant association between DM and AD, and this may be due to confounding by comorbidities such as hypertension and stroke. Therefore, this meta-analysis aimed to systematically investigate the association between DM and the risk of AD by synthesizing evidence from cohort and case–control studies.

## Methods

This meta-analysis was conducted in accordance with the Preferred Reporting Items for Systematic Reviews and Meta-Analyses (PRISMA) guidelines (23–25) and was registered on PROSPERO (CRD420251159844).

### Inclusion criteria

We formulated the PICOS criteria as follows:
P (patients): adults aged ≥18 years, including individuals with AD and non-AD controls(1)
E (exposure): diagnosis of DM(2)
C (comparator): no history of DM(3)
O (outcome): incidence or progression of AD(4)
(5) S (study design): cohort study or case–control study

### Exclusion criteria

1. Duplicated publications
2. Studies lacking sufficient data to calculate HR, odds ratio (OR), relative risk (RR), or their 95% CIs for the association between DM and AD risk
3. Letters, abstracts, case reports, animal studies, conference proceedings, comments, meta-analysis, reviews, and clinical practice guidelines
4. Studies not investigating the association between DM and AD risk

### Search strategy

A computerized search was performed in PubMed, Embase, and Web of Science databases using a combination of MeSH terms and free-text keywords to identify studies on the association between DM and AD risk. The search period was from database inception to October 2025. The search terms included “diabetes mellitus” AND “Alzheimer’s disease” (and their synonyms, such as “type 2 diabetes,” “diabetes,” “Alzheimer disease”) in the title or abstract. Additionally, manual retrieval of the reference lists of the included studies and relevant reviews was conducted to improve recall. The detailed search strategies for each database are presented in **Supplementary Tables S1**-**S3**.

### Data extraction

All retrieved references were imported into EndNote X9 software to remove duplicates. Two independent reviewers screened titles and abstracts against the inclusion and exclusion criteria. Discrepancies were resolved through discussion. The full texts of potentially eligible studies were then reviewed independently by the two reviewers to confirm eligibility and extract data. The extracted information included (1) basic study characteristics (first author, publication year, country, and study design), (2) baseline characteristics of the participants (sample source, sample size, mean age, and gender distribution), (3) diagnostic criteria for AD, (4) outcome measures, effect sizes (HR/OR/RR), and 95% CIs, (5) adjusted confounding factors, (6) follow-up duration, and (7) risk of bias assessment results.

### Bias assessment

We employed the Newcastle–Ottawa Scale (NOS) to assess the risk of bias across the included studies. The scale has a maximum total score of 9 points, with studies scoring 0–3, 4–6, and 7–9 points categorized as low, moderate, and high quality, respectively.

### Statistical analysis

RevMan 5.3 software was used to pool HRs and their 95% CIs to evaluate the association between DM and AD risk. Heterogeneity among studies was assessed using Cochran’s *Q* test and *I*² statistic. A fixed-effects model was used for meta-analysis when heterogeneity was low (*P* ≥ 0.1 and *I*² < 50%), whereas a random-effects model was used when heterogeneity was high (*P* < 0.1 and *I*² ≥ 50%). Subgroup analyses were performed based on sample size (<100,000 vs. >100,000), ethnicity (Asian vs. non-Asian), and adjustment for APOE ϵ4 mutation (yes vs. no). Publication bias was evaluated using Egger’s test, Begg’s test, and funnel plots (for outcomes with ≥10 studies included). Sensitivity analysis was conducted by sequentially excluding each study to assess the stability of the pooled results.

## Results

### Study characteristics

**Figure 1:**
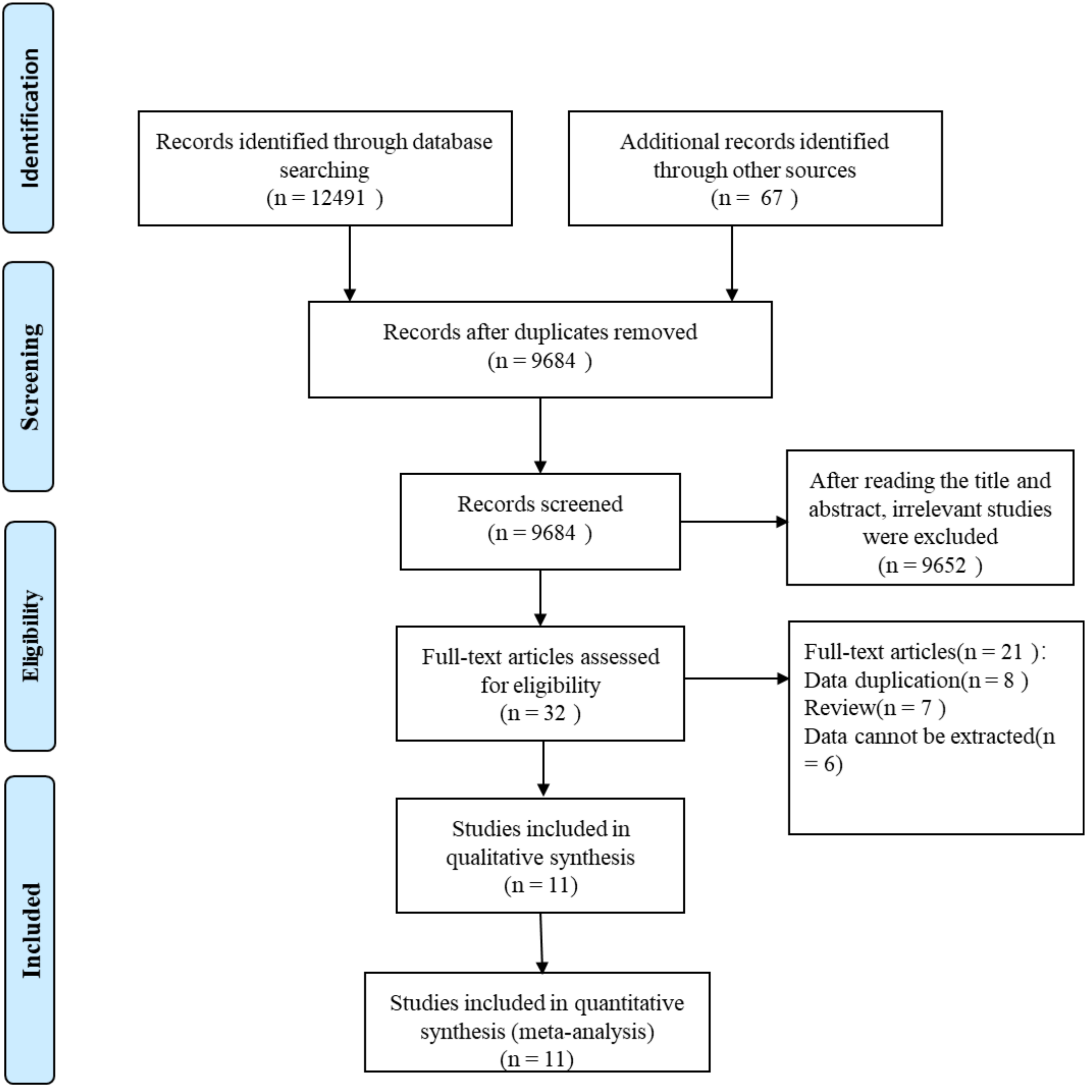
Flowchart of the database search and study inclusion.

A total of 12,558 records were initially retrieved from the databases and other sources. After removing duplicates and screening titles, abstracts, and full texts, 11 studies (26–36) were finally included in the meta-analysis (**Figure 1**). These studies included 10 cohort studies and one case–control study, conducted in the United States, Sweden, Japan, Denmark, South Korea, and China. The sample size of the included studies ranged from 1,855 to 1,259,466, with a total of 3,393,545 participants. The NOS scores of the included studies ranged from 6 to 8, indicating moderate to high quality (**Table 1**). The longest follow-up duration was 20 years. Detailed characteristics of the included studies are summarized in **Table 2**.

**Table 1:** Risk of bias assessment.

Author	Selection	Comparability	Outcome/exposure	Total score	Quality
Akomolafe et al., 2006 (26)	★★	★★	★★	6	Moderate
Dybjer et al., 2023 (27)	★★★	★★	★★	7	High
Huang et al., 2014 (28)	★★★	★★	★★	7	High
Kadohara 2017 (29)	★★	★★	★★	6	Moderate
Leibson et al., 1997 (30)	★★	★★	★★	6	Moderate
Pedersen et al., 2022 (31)	★★★	★★	★★	7	High
Peng et al., 2024 (32)	★★★★	★★	★★	8	High
Ravipati et al., 2022 (33)	★★★	★★	★★	7	High
Seo et al., 2024 (34)	★★★★	★★	★★	8	High
Thomassen et al., 2020 (35)	★★★	★★	★★	7	High
Wang et al., 2025 (36)	★★★★	★★	★★	8	High

One star represents 1 point.

**Table 2:** Summary characteristics and findings of the included studies.

Author (year)	Type of study	Region	Type of population	Sample size (*n*)	Mean age (years)	Gender (male/female)	Diagnostic criteria for Alzheimer’s disease	Adjusted factor	NOS score	Follow-up, year
Akomolafe et al., 2006 (26)	Cohort study	USA	Diabetes mellitus	2,210	70	885/1,325	Criteria from the National Institute of Neurological and Communicative Diseases and Stroke/Alzheimer’s Disease and Related Disorders Association	Age, sex, education, plasma homocysteine, systolic blood pressure, body mass index, current smoking, alcohol use, prevalent stroke, and cardiovascular disease	6	20
Dybjer et al., 2023 (27)	Cohort study	Sweden	Type 2 diabetes	29,139	58.1 (7.61)	11,539/17,600	Diagnostic and Statistical Manual of Mental Disorders, Fifth Edition	Age, sex, education, smoking, alcohol consumption, physical activity level, SBP, BMI, blood pressure medication, lipid lowering treatment, ApoB/ApoA ratio, and history of cardiovascular disease	7	NR
Huang et al., 2014 (28)	Cohort study	China	Diabetes mellitus	142,744	58.7 (14.0)	73,928/68,816	The diagnoses of Alzheimer’s disease were based on history, physical examination, laboratory and imaging studies, the Mini-Mental State Examination, internationally accepted criteria for Alzheimer’s disease (National Institute of Neurological and Communicative Disorders and Stroke–Alzheimer’s Disease and Related Disorders Association), and the Diagnostic and Statistical Manual of Mental Disorders	Age, sex, comorbidities (including hypertension, hyperlipidemia, stroke, coronary artery disease, arrhythmia, heart failure, and depression), geographic area, urbanization status, and medications for diabetes mellitus treatment (including metformin, sulfonylureas, thiazolidinediones, α-glucosidase blockers, non-sulfonylurea insulin secretagouge, and insulin)	7	11
Kadohara 2017 (29)	Case–control study	Japan	Diabetes mellitus	1,855	56.7 (5.38)	965/890	ICD-10	Prescription of antidepressants, antipsychotics, antithrombotics, antihypertensive, and antihyperlipidemic agents and polypharmacy	6	12
Leibson et al., 1997 (30)	Cohort study	USA	Diabetes mellitus	11,436	NR	NR	Diagnostic and Statistical Manual of Mental Disorders, Third Edition	NR	6	15
Pedersen et al., 2022 (31)	Cohort study	Danish	Diabetes mellitus	776,796	NR	NR	ICD-10	Sex, age, depression, marital status, use of antihypertensive drugs, lipid-lowering drugs, and Charlson Comorbidity Index: myocardial infarction, congestive heart failure, cerebrovascular disease, chronic pulmonary disease, connective tissue disease and rheumatologic disease, ulcer disease, mild or moderate-severe liver disease, renal disease, hemiplegia or paraplegia, any malignancies (including leukemia and lymphoma), and acquired immunodeficiency syndrome	7	NR
Peng et al., 2024 (32)	Cohort study	UK	Diabetes mellitus	321,896	57	140,025/181,871	ICD-10	NR	8	NR
Ravipati et al., 2022 (33)	Cohort study	USA	Diabetes mellitus	33,456	72.06 (9.01)	10,513/22,943	The US Alzheimer’s Disease Centers implemented Version 3 of the Uniform Data Set	Gender, race, education, age, congestive heart failure, hypertension, hypercholesterolemia, thyroid disease, vitamin B12 deficiency, atrial fibrillation, heart attack/cardiac arrest, years smoked cigarettes, and alcohol abuse	7	NR
Seo et al., 2024 (34)	Cohort study	Korea	Type 2 diabetes	1,259,466	55.7 (13)	1,090,568/168,898	ICD-10	Severe hypoglycemia	8	6.3
Thomassen et al., 2020 (35)	Cohort study	Denmark	Type 2 diabetes	784,434	65	334,526/449,908	ICD-10	Age, gender, educational level, ethnicity, community size, region, and civil status	7	NR
Wang et al., 2025 (36)	Cohort study	China	Diabetes mellitus	30,113	66.04	13,430/16,683	NR	Age, gender, history of stroke, and heart disease	8	7.29

NOS, Newcastle–Ottawa Scale; NR, not reported.

### Association between DM and the risk of AD

**Figure 2:**
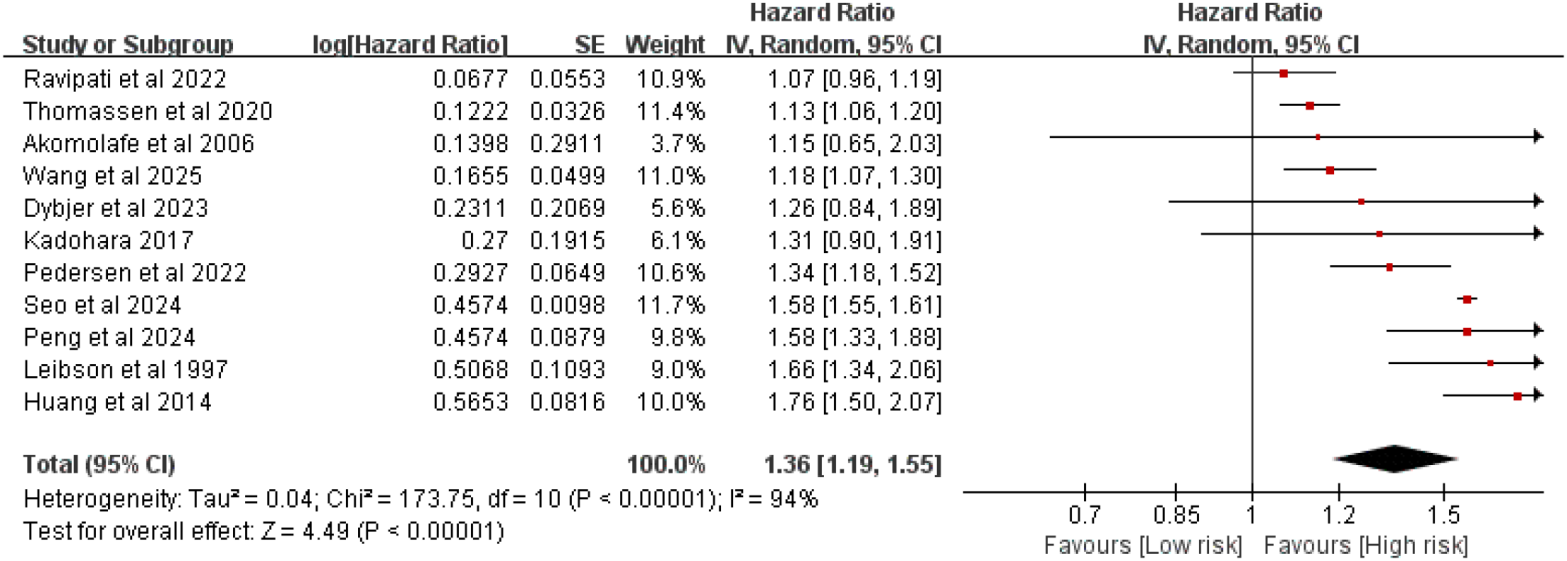
Forest plot of the association between diabetes mellitus and the risk of Alzheimer’s disease.

A total of 11 studies reported an association between DM and AD risk. Heterogeneity analysis showed significant heterogeneity among studies (*I*² = 94%, *P* < 0.00001), so a random-effects model was used for meta-analysis. The results showed that DM was significantly associated with an increased risk of AD (HR = 1.36, 95% CI (1.19–1.55), *P* < 0.00001) (**Figure 2**).

### Subgroup analysis

**Figure 3:**
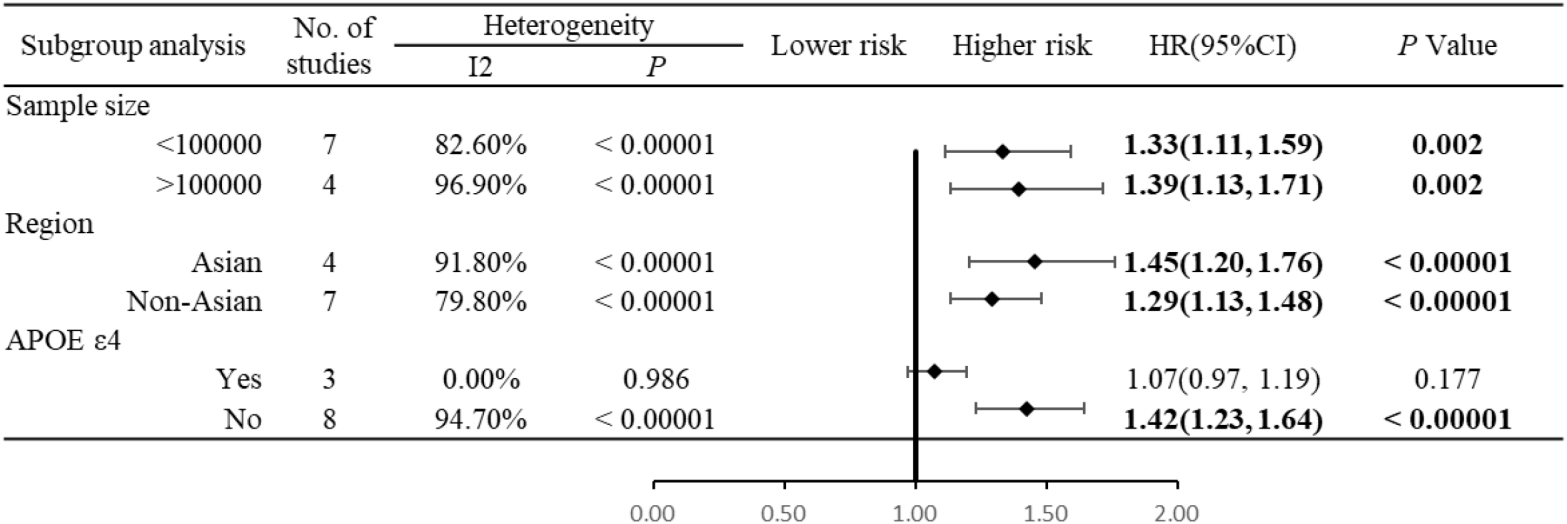
Subgroup analysis forest plot of the association between diabetes mellitus and the risk of Alzheimer’s disease.

Sample size: DM was associated with an increased risk of AD in both small-sample-size (<100,000 participants: HR = 1.33, 95% CI (1.11–1.59), *P* = 0.002) and large-sample-size (>100,000 participants: HR = 1.39, 95% CI (1.13–1.71), *P* = 0.002) subgroups (**Figure 3**).

Ethnicity: The association between DM and AD risk was significant in both Asian (HR = 1.45, 95% CI (1.20–1.76), *P* < 0.00001) and non-Asian populations (HR = 1.29, 95% CI (1.13–1.48), *P* < 0.00001) (**Figure 3**).

Adjustment for APOE ϵ4 mutation: After adjusting for APOE ϵ4 mutation, there was no significant association between DM and AD risk (HR = 1.07, 95% CI (0.97–1.19), *P* = 0.177). In contrast, without adjustment for APOE ϵ4 mutation, DM was significantly associated with an increased AD risk (HR = 1.42, 95% CI (1.23–1.64), *P* < 0.00001) (**Figure 3**).

### Sensitivity analysis

**Figure 4:**
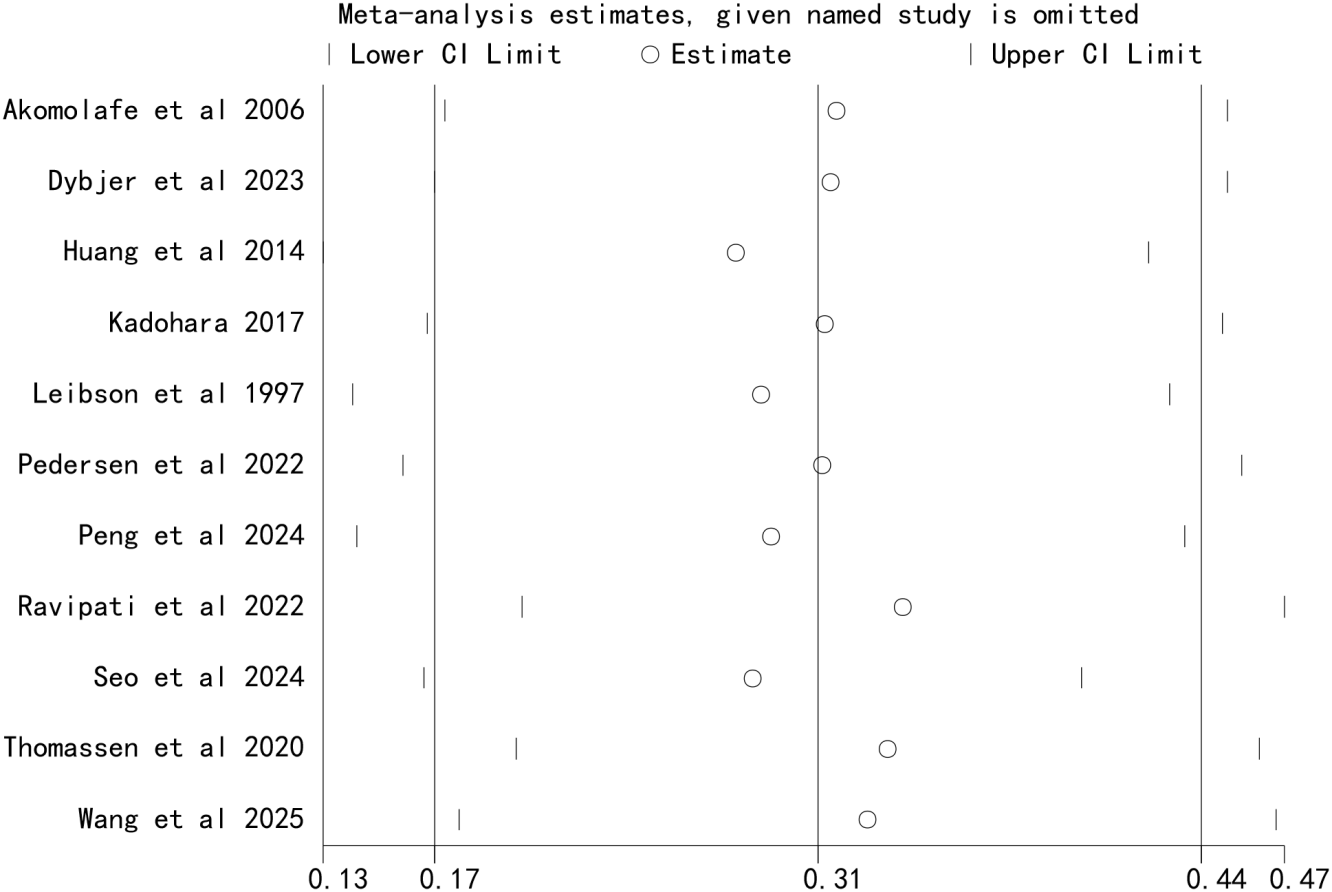
Sensitivity analysis of the association between diabetes mellitus and the risk of Alzheimer’s disease.

Sensitivity analysis was performed by sequentially excluding each study to assess the stability of the pooled results. The results showed that the pooled HR remained consistent after excluding any single study, indicating that the meta-analysis results were stable and not influenced by individual studies (**Figure 4**). Additionally, meta-analysis using a fixed-effects model also showed that DM increased the risk of AD (HR = 1.51, 95% CI (1.48–1.53), *P* < 0.00001). These findings further confirmed the stability of the results.

### Publication bias

**Figure 5:**
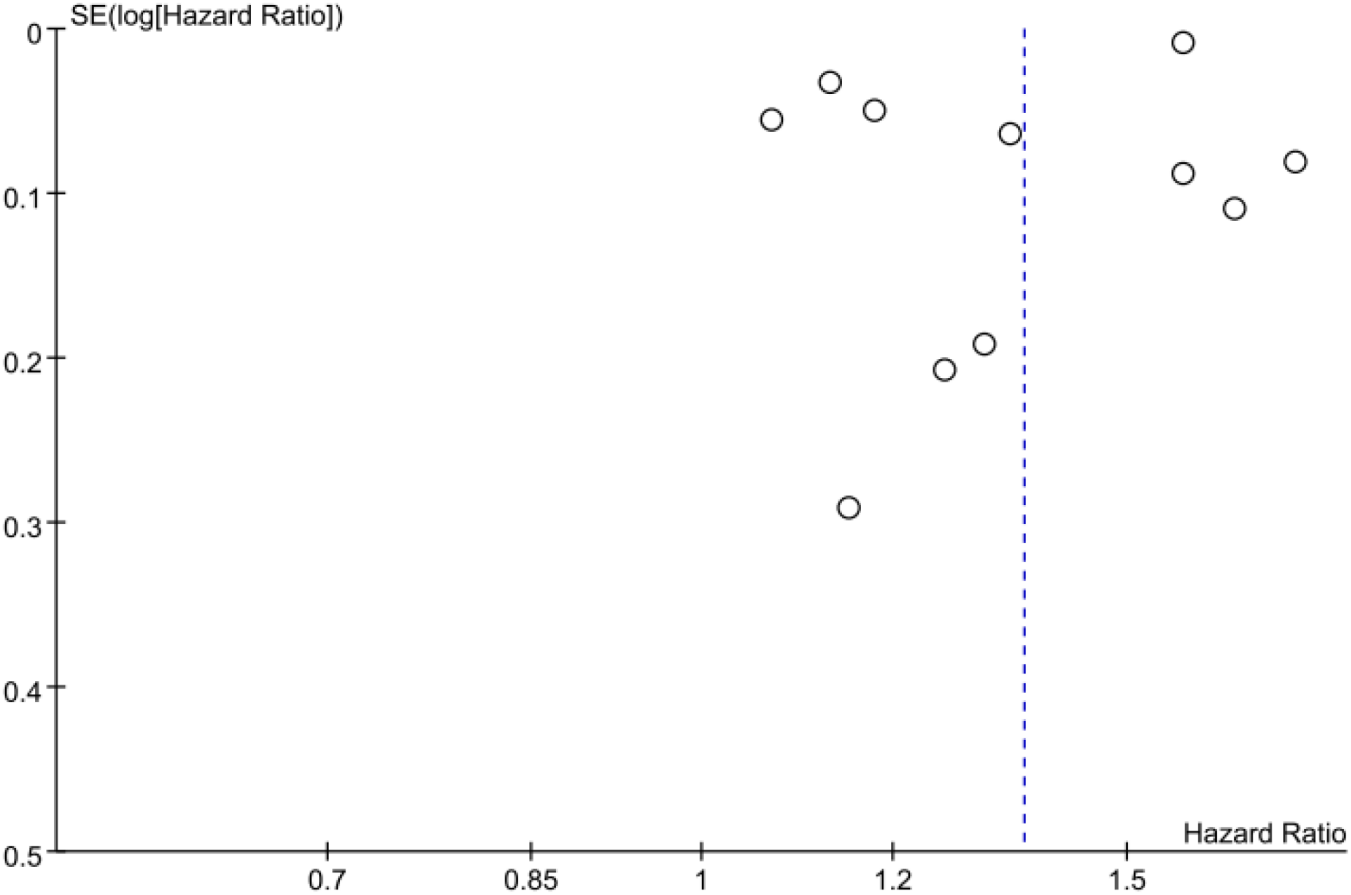
Funnel plot of the association between diabetes mellitus and the risk of Alzheimer’s disease.

Egger’s test (*P* = 0.219) and Begg’s test (*P* = 0.016) suggested potential publication bias. The funnel plot was asymmetric, further indicating the presence of publication bias (**Figure 5**).

## Discussion

This rigorous meta-analysis synthesized evidence from 11 studies involving over 3.3 million participants, confirming a significant positive association between DM and AD risk across diverse populations. The association remained consistent regardless of sample size, indicating that sample size had little impact on the results. The subgroup analysis by ethnicity showed that DM was associated with increased AD risk in both Asian and non-Asian populations, with a slightly stronger effect in Asians. This suggests that ethnicity may moderately influence the association but is not a decisive factor, which may be attributed to differences in genetic background, lifestyle, and environmental factors among ethnic groups—for example, the high-carbohydrate diet in Asian populations may lead to greater blood glucose fluctuations, increasing DM risk and subsequently affecting AD development. Additionally, genetic susceptibility in Asians may make them more vulnerable to the adverse effects of DM on cognitive function.

The APOE ϵ4 allele is the most well-established genetic risk factor for sporadic AD, closely linked to core AD biomarkers (Aβ and tau protein) (37, 38). After adjusting for the APOE ϵ4 allele, the association between DM and AD risk became non-significant. This may be due to the limited availability of APOE ϵ4 genotyping data in only a subset of included studies, which restricted the robustness of the adjusted analysis. The APOE ϵ4 allele promotes AD pathogenesis by regulating Aβ metabolism and clearance, exacerbating neuroinflammation, and inducing tau hyperphosphorylation (39). Pathological processes associated with DM, such as advanced glycation end product (AGE) formation induced by hyperglycemia, oxidative stress, and systemic inflammation, can upregulate brain APOE expression and promote its pathological conformational changes (40). Therefore, we hypothesize that DM may not directly cause AD but acts as an important disease-modifying factor or accelerator. In APOE ϵ4 carriers, DM amplifies pre-existing pathological vulnerability, thereby increasing AD risk; even in the absence of APOE ϵ4, DM-related metabolic disturbances (e.g., insulin resistance, oxidative stress) may independently contribute to AD pathogenesis, although the effect may be weaker (consistent with the significant association observed without APOE ϵ4 adjustment: HR = 1.42, 95% CI (1.23–1.64), *P* < 0.00001). Conversely, in the presence of APOE ϵ4, DM and related metabolic dysregulations (e.g., brain insulin resistance) may synergize with APOE ϵ4 to accelerate Aβ deposition, tau pathology, and neuronal dysfunction, leading to a statistically significant epidemiological association. This explanation is consistent with our findings (significant association without APOE ϵ4 adjustment vs. non-significant association with adjustment) and is supported by previous studies (33). Future research should collect APOE ϵ4 genotyping data in large-scale prospective cohorts and conduct comprehensive adjusted analyses to more reliably evaluate the moderating or mediating role of APOE ϵ4 in the DM–AD association.

Our results are consistent with previous studies (34–36). Insulin resistance and oxidative stress induced by DM can lead to neuroinflammation and Aβ deposition, thereby increasing AD risk (41). This consistency further supports the notion that DM promotes AD onset and progression through multiple pathophysiological pathways. Additionally, Mendelian randomization studies have shown that DM is associated with increased AD risk through genetic predisposition, providing genetic evidence for a causal relationship between DM and AD (42).

DM may increase the risk of AD through multiple mechanisms, with dysfunction of the insulin signaling pathway representing a key contributor (43). In patients with DM, insulin resistance disrupts insulin signaling cascades and impairs neuronal glucose uptake and utilization, leading to energy metabolism dysfunction. This subsequently deprives neurons of adequate energy supply and compromises their physiological function (44). Dysregulated insulin signaling also interferes with the synthesis, release, and metabolism of neurotransmitters, inducing neurotransmitter imbalances that disrupt neuronal communication and precipitate cognitive impairment (45, 46). Oxidative stress constitutes another critical mechanism underlying the promotion of AD pathogenesis by DM. Hyperglycemic conditions trigger excessive generation of reactive oxygen species (ROS), which overwhelms the body’s antioxidant defense systems and initiates a state of oxidative stress (47). Oxidative stress induces lipid peroxidation of neuronal cell membranes and oxidative damage to proteins and nucleic acids, disrupting neuronal structure and function and accelerating neurodegenerative processes. Additionally, it promotes Aβ aggregation and abnormal tau hyperphosphorylation, leading to the formation of neurofibrillary tangles—hallmarks pathological of AD (47, 48).

APOE ϵ4 is one of the strongest genetic risk factors for AD (49). Carriers of APOE ϵ4 allele exhibit an increased propensity for Aβ deposition and neurofibrillary tangle formation, thereby elevating their risk of developing AD (50). Chronic low-grade inflammation is a common feature in patients with DM, characterized by elevated circulating levels of proinflammatory cytokines including tumor necrosis factor-α (TNF-α), interleukin-6 (IL-6), and C-reactive protein (CRP) (51). These proinflammatory mediators can cross the blood–brain barrier (BBB), infiltrate the central nervous system (CNS), and activate microglia and astrocytes, triggering neuroinflammation (52, 53). Neuroinflammation plays a pivotal role in the pathogenesis of AD (54); excessive microglial activation leads to the overproduction and release of proinflammatory cytokines, which further exacerbate neuronal damage and cognitive dysfunction (54, 55). Additionally, neuroinflammation may perturb insulin signal transduction, forming a vicious cycle that exacerbates the pathological progression of both DM and AD (56).

Under the hyperglycemic conditions associated with DM, non-enzymatic glycation reactions occur between glucose and biomacromolecules such as proteins and lipids, leading to the formation of advanced glycation end products (AGEs) (57). AGEs accumulate in the brain parenchyma, where they promote Aβ aggregation and tau hyperphosphorylation, further amplify oxidative stress and neuroinflammation, and induce neuronal injury (58). Notably, metabolic perturbations in DM also disrupt the homeostatic balance between glutamate and gamma-aminobutyric acid (GABA), a key contributor to the development of cognitive impairments (59).

Given the confirmed association between DM and AD, exploring potential interventions to mitigate this risk is clinically relevant. Accumulating evidence suggested that antidiabetic agents may exert potential efficacy on AD. Preclinical and clinical studies have demonstrated that these agents can enhance neuronal survival and ameliorate cognitive dysfunction in AD patients by attenuating Aβ aggregation and abnormal tau hyperphosphorylation, reducing proinflammatory markers, mitigating oxidative stress, and upregulating neurotrophic factor expression (60). Glucagon-like peptide-1 receptor agonists (GLP-1RAs), beyond their well-established role in glycemic control, exert robust neuroprotective properties that slow the progression of neurodegenerative diseases. Specifically, GLP-1RAs effectively inhibit Aβ accumulation and alleviate excessive tau hyperphosphorylation and aggregation during disease progression (61). *In vivo* studies have further revealed the anti-inflammatory effects of GLP-1RAs, which modulate neuroinflammatory responses to reduce neuronal damage and cognitive decline in AD (62, 63). Liraglutide, a representative GLP-1RA, may alleviate cognitive impairment in AD by reducing tau hyperphosphorylation (64). A database analysis indicated that GLP-1RAs and rosiglitazone may reduce the risk of AD in patients with T2DM (65). A recent observational study reported that both sodium-glucose cotransporter-2 inhibitors (SGLT2is) and GLP-1RAs were associated with a reduced risk of AD and related dementias in patients with T2DM, with no significant difference in efficacy observed between the two drug classes (66). GLP-1RA administration was consistently linked to a lower risk of dementia, particularly in the T2DM population (67). A pooled analysis of data from Danish national health registries and cardiovascular outcome clinical trials showed that individuals treated with semaglutide or liraglutide had a dementia risk of less than half that of non-users (67). Another study identified a significant association between semaglutide use and a reduced risk of first-time AD diagnosis (68). Despite these promising observational and pooled analysis findings, however, the 3-year EVOKE and EVOKE+ trials found no significant differences in all primary and secondary cognitive and functional outcomes between the semaglutide and placebo groups (69).

To date, the effects of antidiabetic agents on AD remain under active investigation, and additional well-designed clinical trials are required to validate their therapeutic efficacy and safety profiles in AD patients. Collectively, the association between DM and AD is mediated by a complex interplay of multiple pathophysiological mechanisms, including insulin resistance and signaling dysfunction, chronic neuroinflammation, AGE accumulation, and neurotransmitter imbalance, with antidiabetic agents exhibiting potential modulatory effects on these pathological processes. Future research should focus on further elucidating these underlying mechanisms to develop more effective preventive and therapeutic strategies for AD, particularly in individuals with comorbid DM.

### Limitation

This study has some limitations. First of all, this study only included English literature, which may have language bias. Second, some studies did not fully adjust confounding factors, such as hypertension, hyperlipidemia, obesity, etc., closely related to the occurrence of DM and AD, which may lead to result bias. Third, APOE ϵ4 data was only available in a small subset of studies, limiting the ability to adjust the analysis. Fourthly, there are fundamental differences in the pathophysiological mechanisms between T1DM and T2DM, which may have different effects on the risk of AD. However, the majority of the included studies did not report independent data stratified by DM subtypes or only provided general reports of the overall effect of DM. This prevented us from assessing the potential differences in the impact of T1DM and T2DM on the risk of AD and may have masked important sources of heterogeneity. Future studies should clearly distinguish between DM types and provide stratified risk estimates. Lastly, genetic backgrounds and gene polymorphisms vary among ethnic groups, which may affect susceptibility to DM and AD and increase study heterogeneity.

## Conclusions

This meta-analysis provides strong evidence that DM is an independent risk factor for AD, offering important implications for clinical practice and future research. Clinicians should closely monitor cognitive function in DM patients, detect early cognitive impairment, and implement timely interventions to delay AD onset and progression. However, due to methodological limitations, the results should be interpreted with caution. Future large-scale, high-quality prospective cohort studies are needed to fully elucidate the relationship between DM and AD risk.

## Data Availability

The datasets presented in this study can be found in online repositories. The names of the repository/repositories and accession number(s) can be found in the article/**Supplementary Material**.
