# Supplementary material for: Association between diabetes mellitus and risk of Alzheimer’s disease: a meta-analysis and systematic review
Source: Front Endocrinol (Lausanne). 2026 Mar 3;17:1736410. doi: 10.3389/fendo.2026.1736410 (PMC12991972; doi:10.3389/fendo.2026.1736410)
Supplement: Supplementary file 1 [file DataSheet1.docx]

| **Table S1.Search strategy in PubMed** | |
| --- | --- |
| Search | Query |
| #1 | Search:("Diabetes Mellitus"[Mesh] OR "Diabetes Mellitus, Type 2"[Mesh] OR "Diabetes Mellitus, Type 1"[Mesh] OR "Diabetes Complications"[Mesh] OR "Insulin Resistance"[Mesh] OR "Hyperglycemia"[Mesh] OR diabet*[tiab] OR "type 2 diabetes"[tiab] OR "type 1 diabetes"[tiab] OR "T2DM"[tiab] OR "T1DM"[tiab] OR "non-insulin dependent diabetes"[tiab] OR "insulin dependent diabetes"[tiab] OR "impaired glucose tolerance"[tiab] OR "impaired fasting glucose"[tiab] OR "metabolic syndrome"[tiab]) |
| #2 | Search:("Alzheimer Disease"[Mesh] OR "Dementia"[Mesh] OR "Cognitive Dysfunction"[Mesh] OR "Neurodegenerative Diseases"[Mesh] OR alzheimer*[tiab] OR dementia[tiab] OR "cognitive decline"[tiab] OR "cognitive impairment"[tiab] OR "neurodegenerative"[tiab] OR "senile dementia"[tiab] OR "cognitive disorder*"[tiab] OR "memory disorder*"[tiab]) |
| #3 | Search:("Cohort Studies"[Mesh] OR "Case-Control Studies"[Mesh] OR "Longitudinal Studies"[Mesh] OR "Prospective Studies"[Mesh] OR "Retrospective Studies"[Mesh] OR cohort[tiab] OR "case control"[tiab] OR longitudinal[tiab] OR prospective[tiab] OR retrospective[tiab] OR "follow-up"[tiab] OR "incidence"[tiab] OR "risk"[tiab]) |
| #4 | Search:(#1) AND (#2) AND (#3) |
| **Table S2.Search strategy in Web of Science** | |
| Search | Query |
| #1 | Search:(diabet* OR "type 2 diabetes" OR "type 1 diabetes" OR T2DM OR T1DM OR "non-insulin dependent diabetes" OR "insulin dependent diabetes" OR "impaired glucose tolerance" OR "impaired fasting glucose" OR "metabolic syndrome") |
| #2 | Search:(alzheimer* OR dementia OR "cognitive decline" OR "cognitive impairment" OR neurodegenerative OR "senile dementia" OR "cognitive disorder*" OR "memory disorder*") |
| #3 | Search:(cohort OR "case control" OR longitudinal OR prospective OR retrospective OR "follow-up" OR incidence OR risk) |
| #4 | Search:(#1) AND (#2) AND (#3) |
| **Table S3.Search strategy in Embase** | |
| Search | Query |
| #1 | Search:(exp diabetes mellitus/ or exp type 2 diabetes/ or exp type 1 diabetes/ or exp diabetic complication/ or exp insulin resistance/ or exp hyperglycemia/) |
| #2 | Search:(diabet* or "type 2 diabetes" or "type 1 diabetes" or T2DM or T1DM or "non-insulin dependent diabetes" or "insulin dependent diabetes" or "impaired glucose tolerance" or "impaired fasting glucose" or "metabolic syndrome").ti,ab,kw. |
| #3 | Search:(#1) OR (#2) |
| #4 | Search:(exp Alzheimer disease/ or exp dementia/ or exp cognitive defect/ or exp neurodegenerative disease/) |
| #5 | Search:(alzheimer* or dementia or "cognitive decline" or "cognitive impairment" or neurodegenerative or "senile dementia" or "cognitive disorder*" or "memory disorder*").ti,ab,kw. |
| #6 | Search:(#4) OR (#5) |
| #7 | Search:(exp cohort analysis/ or exp case control study/ or exp longitudinal study/ or exp prospective study/ or exp retrospective study/) |
| #8 | Search:(cohort or "case control" or longitudinal or prospective or retrospective or "follow-up" or incidence or risk).ti,ab,kw. |
| #9 | Search:(#7) OR (#8) |
| #10 | Search:(#3) AND (#6) AND (#9) |
